# Explainable and uncertainty-aware ensemble framework with causal analysis for breast cancer detection

**DOI:** 10.3389/fonc.2025.1751090

**Published:** 2026-02-20

**Authors:** Muhammad Zaheer Sajid, Muhammad Fareed Hamid, Imran Qureshi

**Affiliations:** 1 Department of Electrical and Computer Engineering, George Mason University, Fairfax, VA, United States; 2 Department of Computer Software Engineering, Military College of Signals, National University of Sciences and Technology, Islamabad, Pakistan; 3 College of Computer and Information Sciences, Imam Mohammad Ibn Saud Islamic University (IMSIU), Riyadh, Saudi Arabia

**Keywords:** breast cancer prediction, causal interpretability, clinical decision support, ensemble learning, SHAP explainability, uncertainty quantification

## Abstract

Breast cancer is one of the main causes of cancer deaths around the world and is known for its aggressive growth and ability to spread. While machine learning has shown good results for diagnosis, most existing methods do not handle uncertainty or explain their predictions clearly. In this study, we present an integrated framework that combines uncertainty-aware ensemble learning with causal feature analysis and multimodal explainability for breast cancer prediction. The framework uses a mix of Light Gradient Boosting Machine (LightGBM), random forest, and gradient boosting classifiers that include uncertainty estimation so that the model can mark predictions that are less confident. It also applies causal analysis to detect possible clinical confounders and uses SHAP (Shapley Additive Explanations), permutation importance, and feature attribution for interpretation. Tests on two public datasets showed strong and consistent performance. On the UCTH Clinical Dataset, the model reached an area under the curve (AUC) of 0.97%, an accuracy of 0.95%, and an F1 score of 0.94%, with 100% precision for high confidence cases and no false positives. On the Breast Cancer Wisconsin dataset, it achieved an AUC of 0.99, an accuracy of 0.94%, and an F1 score of 0.92%, which increased to 0.98% accuracy and 0.98% F1 score when only certain predictions were considered. Causal analysis pointed out important clinical confounders like lymph node involvement, tumor size, and metastasis, while fairness tests showed balanced results across demographic groups. Overall, the framework combines uncertainty estimation and causal interpretability to give predictions that are both accurate and trustworthy. It provides clinicians with clear confidence levels for every prediction and supports transparent decision-making that can reduce diagnostic errors and improve reliability in clinical use.

## 1 Introduction

Breast cancer is a severe disease. It happens when abnormal cells in the breast grow out of control (1). It is the second most common cancer found in women after skin cancer. In 2022, approximately 2.3 million women in the world were diagnosed with breast cancer, and approximately 670,000 died from it (2). Primarily, it affects women, but in some rare cases, men can also have breast cancer. According to abnormal cell growth, breast cancer is divided into two types: invasive and non-invasive (3).

The main risk factors of breast cancer include aging, genetic hereditary mutation, hormonal changes, lifestyle habits, and environmental factors (2). If not treated, the disease spreads to other organs and causes multiple organ failure and death (4). Early detection increases the survival rate of patients (1–3). The common symptoms of breast cancer are swelling or a lump in the breast, chest, or underarm, bloody discharge from the nipple, continuous pain in the breast, and a change in the size or shape of the breast (5).

Mammography is the most common screening method for breast cancer. It helps to find breast density, calcifications, structural problems, and tumor masses (6). Other diagnostic tests include magnetic resonance imaging (MRI), positron emission tomography (PET) scans, computed tomography (CT) scans, biopsy, gene testing, and estrogen or progesterone receptor tests. Treatment methods include surgery, chemotherapy, radiation therapy, hormone therapy, targeted therapy, and immunotherapy (4). Humans cannot control genetic factors, but a healthy lifestyle can reduce the chances of breast cancer (7). The preventive steps include less use of alcohol and smoking, keeping body weight normal, doing regular exercise, breastfeeding, and not using hormone therapy after menopause.

Even with advanced diagnostic technologies, some problems persist, including low imaging resolution, the inability to detect minor symptoms, high procedure costs, and slow diagnosis results (8). According to the National Institutes of Health (NIH), human error is the main reason for almost 96.3% of diagnostic failures (9). The use of machine learning (ML) can solve many of these problems. ML can learn patterns from a large amount of data, and it is helpful for disease prediction and early detection. Many studies show that ML can help doctors diagnose breast cancer correctly. By checking medical data, ML can improve medical decisions, reduce human mistakes, and make diagnoses and treatments faster (9).

Explainable artificial intelligence (XAI) is the method that makes ML results easy to understand for humans (10). XAI not only checks model predictions but also enhances the reliability, stability, and transparency of models, helping to identify and correct errors. Much research has been done on the use of ML and XAI for breast cancer detection and classification. One research used the Wisconsin Diagnostic Breast Cancer (WDBC) dataset, which has 569 records with 32 features, and compared three ML algorithms for breast cancer detection. The k-Nearest Neighbors (kNN) algorithm achieved the highest accuracy of 95.9%, which demonstrates its strong performance in classification (11). Another research used the same dataset but focused on five main features and found that support vector classifier (SVC) gave better result with 93% accuracy (12). One more research used the Mendeley dataset, which has 400 Indonesian patient cases, in which 200 were patients with breast cancer, and found that the XGBoost model gave 85% accuracy. The SHAP (Shapley Additive Explanations) and CRISP MLQ frameworks were used to make ML results more understandable and to check the quality of the model (13).

An ensemble model that used support vector machine (SVM) and random forest (RF) algorithms got a very high accuracy of 99.99% (14). Another research, based on data from 500 patients at Dhaka Medical College Hospital in Bangladesh, used five ML algorithms and found that XGBoost yielded the best performance with 97% accuracy. The use of SHAP made the model clearer and increased trust in the results (15). One additional study utilized a pre-trained ResNet50 model to classify breast tumor images as benign or malignant, achieving 96.84% accuracy. The dataset of this study was taken from the Kaggle repository, and it has 7,909 breast tumor images of 82 patients collected through surgical open biopsy (16).

Despite the notable progress of ML and XAI techniques in breast cancer diagnosis, a critical methodological gap remains. Most existing ML/XAI approaches primarily focus on improving predictive accuracy and providing *post-hoc* explanations, while neglecting three key aspects required for reliable clinical deployment. First, current models rarely incorporate explicit uncertainty estimation, resulting in overconfident predictions that fail to indicate when model outputs may be unreliable. Second, widely used explanation techniques, such as SHAP and LIME, are primarily correlation-based and do not distinguish between causal clinical factors and spurious associations, thereby limiting their ability to identify proper diagnostic drivers and potential confounders. Third, fairness assessment across demographic or temporal patient subgroups is often overlooked, raising concerns about biased or inconsistent performance in real-world settings. Importantly, these limitations are typically addressed in isolation, if at all, and no unified framework jointly integrates uncertainty awareness, causal reasoning, and fairness analysis within a single diagnostic pipeline. This gap restricts the clinical trustworthiness, transparency, and ethical applicability of existing breast cancer prediction systems.

To address these problems, this research presents a new Uncertainty-Aware Causal Explainable Ensemble Framework for breast cancer prediction. The proposed method integrates uncertainty estimation, causal feature analysis, and multimodal explainability into a single ensemble model. By giving predictions with information of when the model is not sure, the framework improves the reliability, understanding, and fairness of diagnosis, and makes a base for trusted clinical decision support in real healthcare use.

### 1.1 Research motivation

Breast cancer is still one of the biggest health problems in the world, and every year, many people die from it despite the fast progress in diagnostic imaging and molecular testing. Modern diagnostic methods like mammography MRI and histopathological test give useful information, but their performance is limited because of human mistakes, high cost, and less availability in low-resource areas. Because of this, there is a growing need for automatic and intelligent diagnostic systems that can help doctors detect cancer fast and correctly. ML and XAI are new solutions that can learn complex clinical patterns from data and give understandable predictions to support medical decision-making.

Nevertheless, a significant gap persists between model accuracy and clinical reliability. Existing ML models often exhibit overconfidence in uncertain scenarios, lack mechanisms to convey prediction reliability, and ignore causal relationships among medical features. These shortcomings undermine the trust, clarity, and ethical deployment of models in real clinical settings. Moreover, many predictive models fail to assess fairness across different patient groups, risking biases related to age, ethnicity, or timing of diagnosis. To foster trust in artificial intelligence (AI) for healthcare, predictive systems must not only prioritize accuracy but also incorporate uncertainty estimation, causal reasoning, interpretability, and fairness assessment.

Driven by these gaps, this research aims to develop an integrated diagnostic framework that bridges advanced ensemble learning with clinical accountability. The proposed Uncertainty-Aware Causal Explainable Ensemble Framework is designed to deliver not only high-accuracy predictions but also explicit uncertainty indicators, interpretable explanations, and fair performance across all patient subgroups. By embedding uncertainty quantification and causal feature analysis into the prediction pipeline, this work seeks to narrow the divide between algorithmic performance and clinical trust, paving the way for reliable, transparent, and ethically aligned AI systems in breast cancer prediction.

### 1.2 Research contribution

This research presents an integrated uncertainty-aware and causally explainable ensemble framework for breast cancer prediction that focuses on diagnostic reliability and clinical transparency. The main contributions are as follows:

1. Developed a mixed ensemble of Light Gradient Boosting Machine (LightGBM), RF, and gradient boosting models to calculate epistemic uncertainty and produce reliable and qualified predictions.
2. Added causal inference by using Cramer’s *V* and point biserial correlation to find confounding clinical factors and make sure the feature relations are causally correct.
3. Combined SHAP values’ permutation importance and model-based importance to give a clear and multi-view understanding of diagnostic predictions.
4. Used bootstrap-based confidence intervals and fairness difference metrics to check model stability and fair performance for different demographic groups.
5. The proposed research achieved 0.95 area under the curve (AUC) and 97.0% accuracy on certain predictions with 100% precision, which makes a clinically reliable and trusted diagnostic support system.

Overall, this research reduces the gap between prediction accuracy and clinical trust by combining uncertainty calculation causal reasoning and fairness-based explainability in one diagnostic AI framework.

## 2 Dataset description

The experimental testing was carried out by using two datasets: one is the UCTH Breast Cancer Clinical Dataset (17) and the other is the Breast Cancer Wisconsin Diagnostic Dataset (18). The use of these two datasets gives both clinical and morphological validation of the proposed framework.

### 2.1 Clinical dataset

The Breast Cancer Clinical Dataset is a structured table dataset that includes demographic pathological and treatment-related features important for breast cancer diagnosis. The dataset has a total of 213 patient records collected from verified clinical sources, and each record is labeled with a binary value that shows the presence (malignant = 1) or absence (benign = 0) of cancer.

### 2.2 Data composition

**Figure 1:**
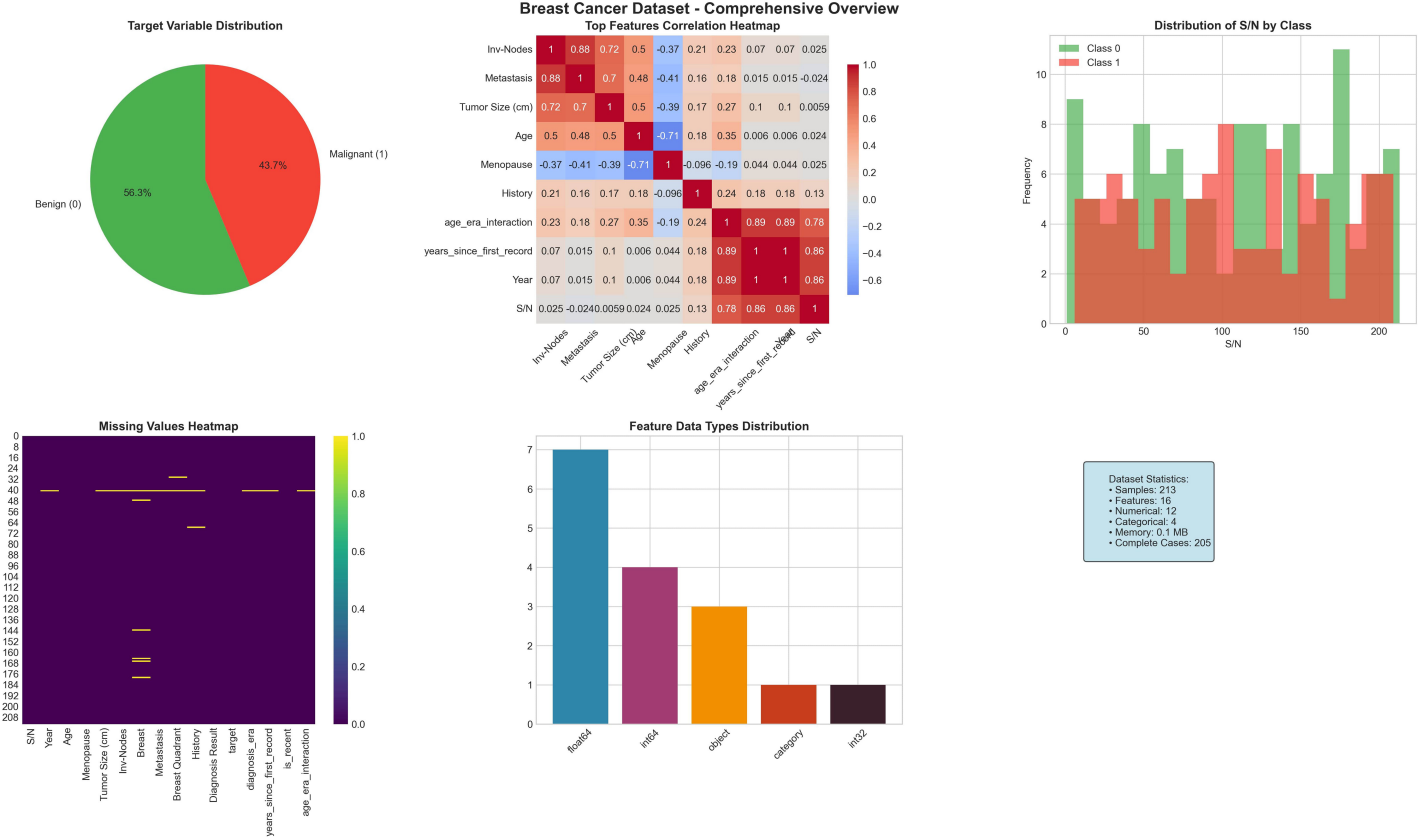
UCTH Breast Cancer dataset comprehensive overview.

**Figure 2:**
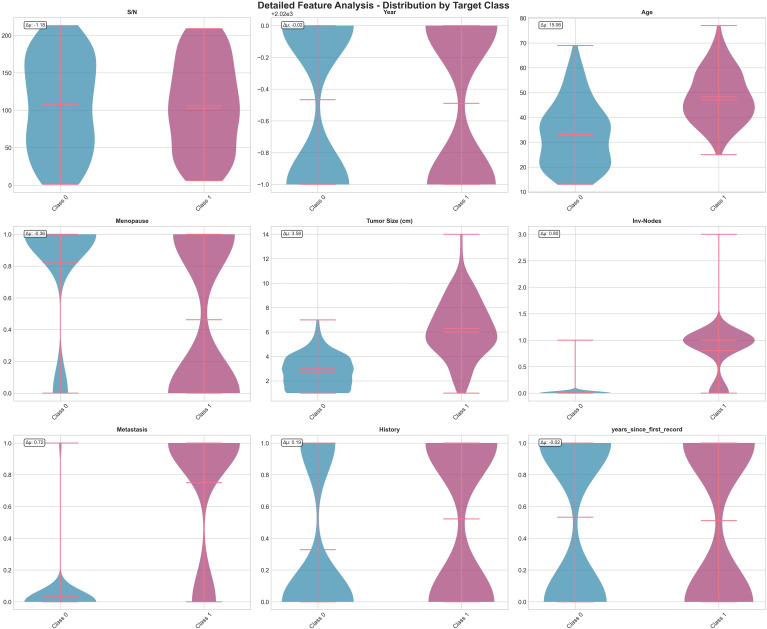
UCTH Breast Cancer dataset detailed feature analysis.

The dataset has 16 main features that show tumor characteristics, lymph node conditions, and patient demographics. After preprocessing and adding time-related features, the total features increased to 20 predictors. **Table 1** provides an overview of the key attributes. **Figure 1** shows the UCTH Breast Cancer dataset comprehensive overview. **Figure 2** shows the UCTH Breast Cancer dataset detailed feature analysis.

**Table 1:** Overview of breast cancer clinical dataset attributes.

Category	Features	Type
	Age, Age_bin, Menopause	Num./Cat.
Demo.	Tumor Size, Inv-Nodes, Node-Caps	Num./Cat.
	Deg-Malig (Grade)	Ord.
Tumor Char.	Breast, Breast-Quad	Cat.
	Irradiation, History	Bin.
Clin. Hist. Tempora	Diagnosis Era, Recency, Age–Era Int.	Der. Num.

*Target:* Class (0 = Benign, 1 = Malignant).

There was a total of 213 samples, of which 120 (56.3%) were benign and 93 (43.7%) were malignant. The dataset was randomly divided into training (70%, *n* = 149) and testing (30%, *n* = 64) parts with the same class ratio. The training data have 84 benign and 65 malignant samples, and the testing data have 36 benign and 28 malignant samples.

### 2.3 Preprocessing and quality assurance

Data preprocessing included filling missing values by using the statistical methods’ mean for numerical variables and mode for categorical variables. All categorical variables were converted by one-hot encoding and numerical features were scaled to zero mean and unit variance. Time-related feature addition was performed to make the dataset richer with attributes like diagnostic era and recency, which show clinical change with time.

### 2.4 Benchmark dataset: Breast Cancer Wisconsin (Diagnostic)

**Figure 3:**
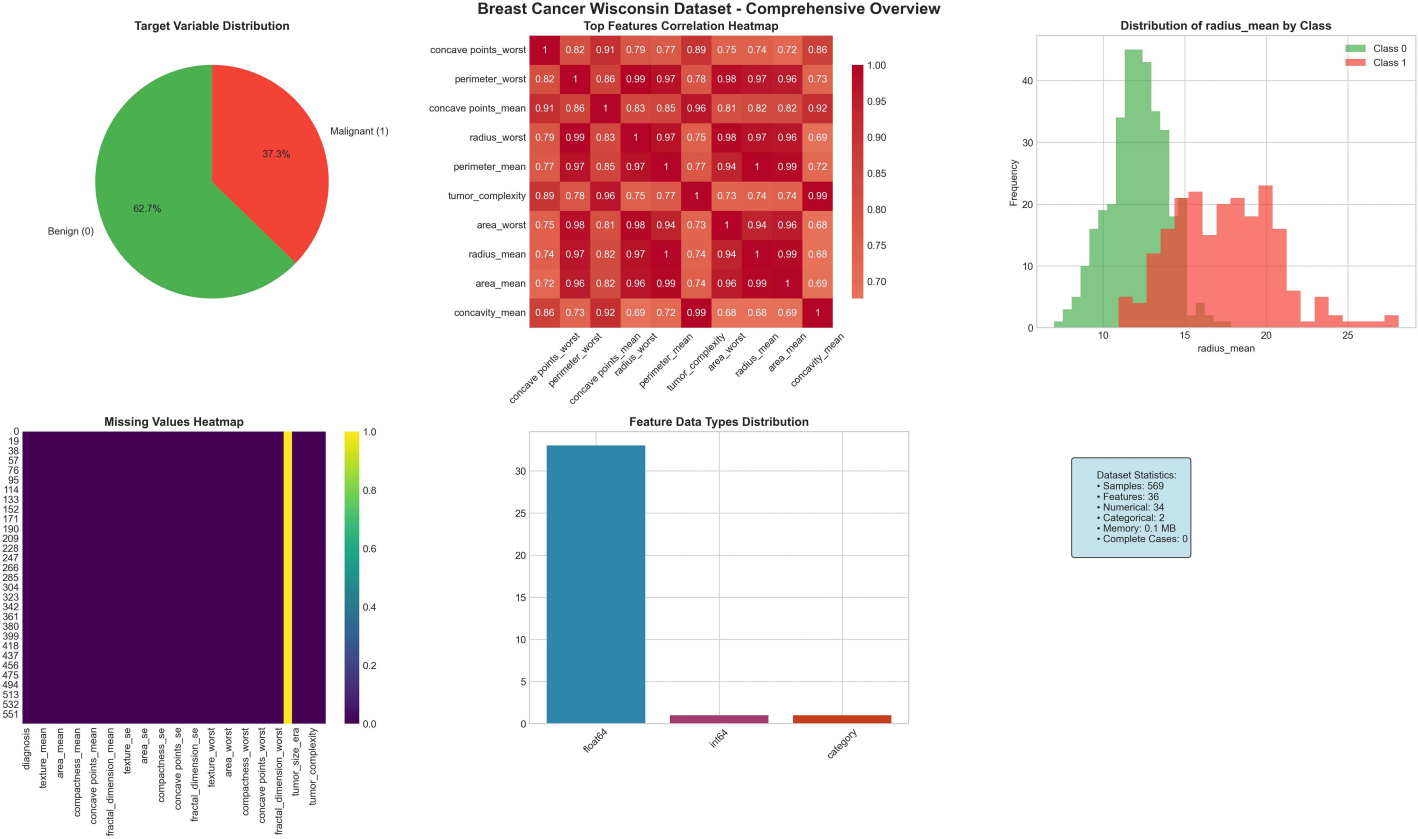
Breast Cancer Wisconsin dataset comprehensive overview.

**Figure 4:**
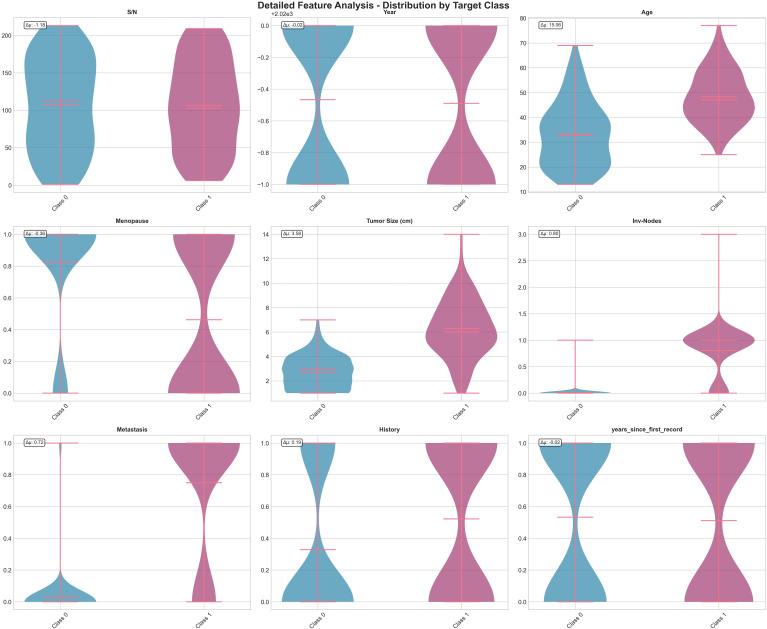
Breast Cancer Wisconsin dataset detailed feature analysis.

Along with the clinical dataset, this research also used the Breast Cancer Wisconsin Diagnostic dataset, which is a public benchmark dataset mostly used for breast cancer classification. It has 569 biopsy samples taken from digital fine needle aspirate (FNA) images of breast tissue, and each sample is labeled as malignant (M) or benign (B). The dataset has 30 numerical features that describe the shape and structure of cell nuclei, like radius, texture, perimeter, area, compactness, concavity, and symmetry calculated with three statistical forms: mean, standard error, and worst values. These attributes capture both global and localized shape irregularities and support strong morphological analysis of tumor malignancy as summarized in **Table 2**. **Figure 3** shows the Breast Cancer Wisconsin dataset comprehensive overview. **Figure 4** shows the Breast Cancer Wisconsin dataset detailed feature analysis.

**Table 2:** Overview of breast cancer Wisconsin (diagnostic) dataset attributes.

Category	Feature examples	Count/Type
Mean features	Radius_mean, Texture_mean, Area_mean	10/Num.
SE features	Radius_se, Texture_se, Compactness_se	10/Num.
Worst features	Radius_worst, Area_worst, Concavity_worst	10/Num.
Target variable	Diagnosis (M = malignant, B = benign)	1/Cat.
Total samples	569 (357 benign, 212 malignant)	–

All features are continuous except for the diagnostic label, and the data are clean, well-normalized, and ready for supervised learning. This dataset supports the clinical dataset by adding high-resolution morphological data that improve model generalization and help in cross domain diagnostic validation.

### 2.5 Ethical and clinical considerations

Both datasets have fully anonymous patient information and follow ethical and data protection rules suitable for secondary research. The clinical dataset was taken from open access medical sources and the proposed method can also be used with institutional datasets after ethical approval. The selected features especially Tumor Size, Inv-Nodes, and Menopausal Status are closely related to standard oncological indicators, which keep the developed framework clinically relevant and understandable.

Overall, the combined use of these datasets makes a balanced and clinically representative base for testing the proposed Uncertainty-Aware Causal Explainable Ensemble Framework. The clinical dataset gives real-world contextual features and the Wisconsin dataset adds strong morphological accuracy; together, they provide complete testing of performance uncertainty and explainability under different clinical data conditions.

## 3 Proposed methodology

**Figure 5:**
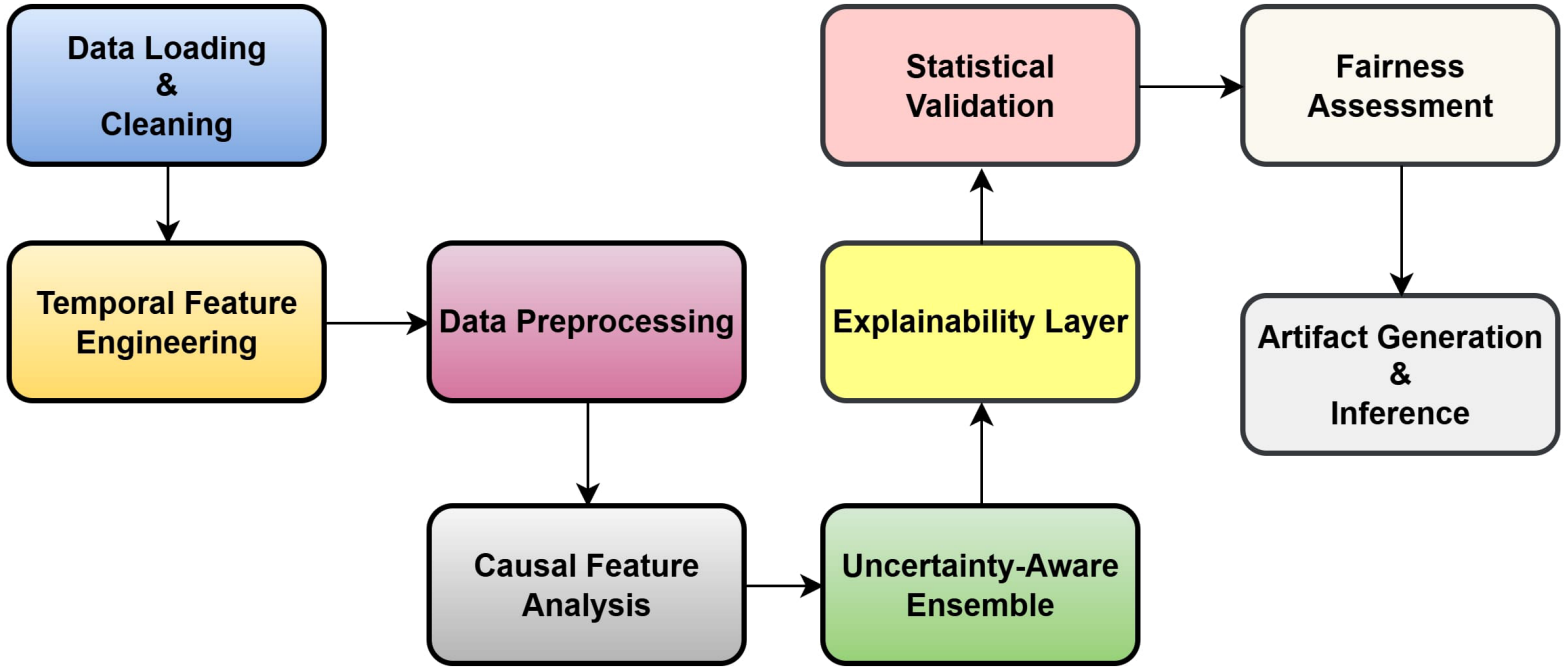
Workflow of the proposed uncertainty-aware causal–explainable ensemble framework for breast cancer prediction.

This research presents an Uncertainty-Aware Causal Explainable Ensemble Framework for breast cancer prediction, which includes uncertainty calculation causal feature analysis multimodal explainability and fairness checking in one reproducible structure. **Figure 5** shows the complete workflow of the proposed framework, which includes the step-by-step process of preprocessing feature engineering model development uncertainty estimation causal analysis and fairness evaluation.

The methodology started with data preprocessing in which the clinical breast cancer dataset was checked for consistency and completeness. Missing values were filled by using statistical estimators; categorical variables were changed by one-hot encoding and numerical features were standardized for balanced scaling. To include time-based changes, a Temporal Feature Engineering module created extra features like diagnosis era years since first record and age–era interaction that help the model learn from long-term diagnostic variations and time-based clinical trends.

An Uncertainty Aware Ensemble Model was developed, which used three different classifiers—LightGBM, RF, and gradient boosting—to take advantage of different learning biases. Each classifier gave probability outputs, and these outputs were combined to calculate the mean predictive probability of all models. The standard deviation of these probabilities was used to measure epistemic uncertainty, which shows the disagreement between ensemble models. Predictions with uncertainty higher than threshold (*τ* = 0.15) were marked as uncertain to produce qualified predictions that focus on reliability more than coverage.

For the ensemble, we chose LightGBM, RF, and gradient boosting because they provide complementary strengths and work well on small to medium clinical tabular datasets. RF reduces variance, gradient boosting learns error correcting patterns, and LightGBM offers fast histogram-based learning with strong support for mixed feature types. These models also produce stable probability outputs, which help with uncertainty estimation and SHAP-based interpretability. We tested other models like XGBoost and simple neural networks, but XGBoost showed higher variance and weaker uncertainty calibration, while neural networks required more data and tuning. The three selected models offered the best balance of accuracy, stability, interpretability, and efficiency, making them suitable for clinical deployment.

To find statistically important and causally related variables, a Causal Feature Analyzer was used, which measured the relation between input features and diagnostic results by using Cramer’s *V* for categorical features and point biserial correlation for continuous features. This analysis showed that Invasive Nodes (Inv Nodes) and Tumor Size are the main clinical factors that affect malignancy. A Robust Multimodal Explainer was also added to improve interpretability and clarity by combining information from SHAP permutation importance and model feature importance. When SHAP calculation was not possible, a permutation-based backup was used to keep interpretability working and reliable for all samples.

Model reliability and generalization were tested by using bootstrap-based confidence interval estimation with 500 to 1,000 iterations for performance measures like accuracy, precision, recall, F1 score, and AUC. A Fairness Assessment Module checked predictive equality for different demographic groups like age and diagnostic era and measured the difference in performance to make sure that the model works fairly. Overall, this framework combines ensemble learning with causal and explainable AI ideas to achieve prediction performance interpretability and fairness suitable for clinical use.

## 4 Proposed architecture

**Figure 6:**
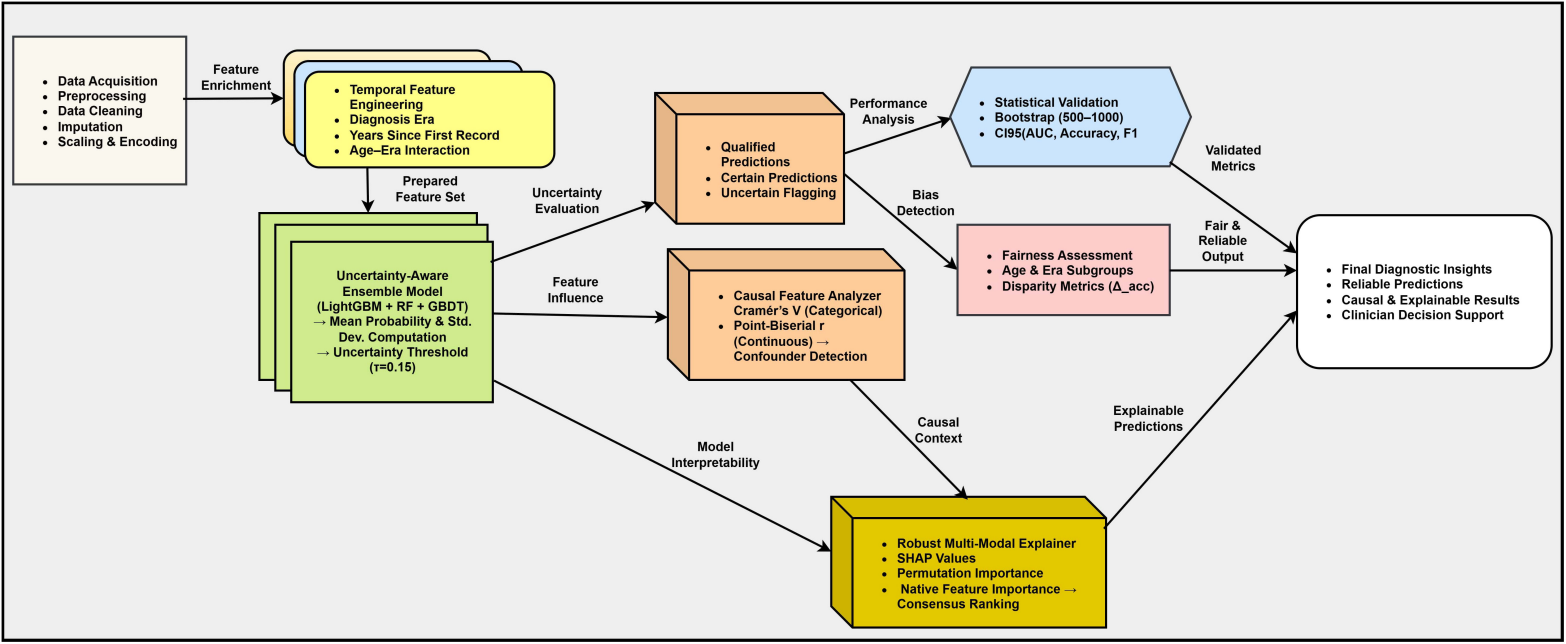
Proposed architecture of the Uncertainty-Aware Causal–Explainable Ensemble Framework for breast cancer prediction. The architecture combines preprocessing temporal feature engineering ensemble uncertainty calculation causal explainability fusion and fairness checking in one diagnostic pipeline.

The proposed architecture is a modular uncertainty-aware and causally explainable ensemble designed to provide diagnostic accuracy and clinical trust. As shown in **Figure 6**, the framework has five connected layers: (1) Data preprocessing and feature engineering, (2) Base ensemble learning, (3) Uncertainty quantification layer, (4) Causal explainability layer, and (5) Statistical fairness validation. Each part gives its own function to make a trusted AI-based breast cancer prediction system. The system begins with a structured dataset D:

(1) $$D={\left\{(x_{i},y_{i})\right\}}_{i=1}^{N},$$

where $x_{i}\in {\mathbb{R}}^{d}$ is the *i*th input vector containing demographic, pathological, and clinical features, and $y_{i}\in \{0,1\}$ represents the ground truth label (0 = benign, 1 = malignant). Data normalization is applied to maintain statistical consistency:

(2) $$x^{\prime }=\frac{x-\mu }{\sigma },$$

where *µ* and *σ* denote the mean and standard deviation of each feature, respectively.

1. Temporal feature augmentation: To model diagnostic changes, a temporal encoder creates time-based attributes that represent diagnosis period recency and the relation between age and diagnostic period. These features help the model to learn generational and time-dependent changes in breast cancer characteristics.

2. Heterogeneous ensemble learning: The model ensemble consists of three classifiers—LightGBM, RF, and gradient boosting—chosen for their complementary learning biases and robustness to clinical data heterogeneity. Each base learner *M_k_* outputs a class probability $p_{k}(y=1|x_{i})$ for the *i*th sample. The ensemble mean probability and epistemic uncertainty are computed as:

(3) $${\overset{¯}{p}}_{i}=\frac{1}{K}\sum_{k=1}^{K}p_{k}(y=1|x_{i}),$$

(4) $$u_{i}=\sqrt{\frac{1}{K}\sum_{k=1}^{K}{\left(p_{k}(y=1|x_{i})-{\bar{p}}_{i}\right)}^{2},}$$

where *K* = 3 denotes the number of models. The final prediction rule incorporates an uncertainty threshold *τ*:

(5) $${\hat{y}}_{i}=\{\begin{array}{ll} 1, & \text{if }{\bar{p}}_{i}\geq 0.5\text{ and }u_{i}\leq \tau ,\\ 0, & \text{if }{\bar{p}}_{i}<0.5\text{ and }u_{i}\leq \tau ,\\ \text{uncertain}, & \text{if }u_{i}>\tau . \end{array}$$

This mechanism ensures that predictions marked as *uncertain* are deferred for clinical review, safeguarding against overconfident misclassifications.

3. Causal feature analysis: To enhance interpretability, causal inference techniques identify confounding variables that may distort model learning. Categorical relationships are quantified using Cramer’s *V*:

(6) $$V=\sqrt{\frac{\chi^{2}/n}{\min(k-1,r-1)}},$$

while continuous–binary associations are captured by the point-biserial coefficient:

(7) $$r_{pb}=\frac{{\bar{x}}_{1}-{\bar{x}}_{0}}{s_{x}}\sqrt{\frac{n_{1}n_{0}}{n^{2}}}.$$

Features with |*r_pb_*|*>* 0.1 or *V >* 0.1 are considered potential confounders, reflecting significant diagnostic influence.

4. Multimodal explainability integration: This layer fuses diverse interpretability techniques SHAP, permutation importance, and model-native feature importance to deliver consistent, clinician-understandable insights. The SHAP contribution for each feature *x_j_* is expressed as:

(8) $$\phi_{j}=E_{S\subseteq F\backslash \{j\}}[\;f_{S\cup \{j\}}(x_{S})-f_{S}(x_{S})],$$

and permutation-based relevance as:

(9) $$I_{j}^{perm}={\text{AUC}}_{baseline}-{\text{AUC}}_{permuted(j)}$$

The consensus importance metric integrates all three views:

(10) $$I_{j}^{final}=\frac{1}{3}(I_{j}^{tree}+I_{j}^{perm}+|\phi_{j}|)$$

5. Statistical and fairness validation: The reliability of the ensemble is established using bootstrap-based confidence intervals:

(11) $$CI_{95}(M)=[P_{2.5}(M^{*}),P_{97.5}(M^{*})],$$

where *M*^∗^ represents bootstrap-resampled metric estimates. Fairness is evaluated by comparing subgroup performance:

(12) $${\text{Δ}}_{acc}(s)=\underset{g\in G_{s}}{\max}\;Acc_{g}-\underset{g\in G_{s}}{\min}\;Acc_{g},$$

ensuring equitable outcomes across demographic groups such as age and diagnostic era.

The proposed architecture makes a closed-loop diagnostic intelligent system where uncertainty interpretability and fairness are the main design principles. Each module helps to produce clinically meaningful and confidence-aware predictions that follow the needs of trusted AI- and evidence-based medical decision-making.

The integration of these parts allows the framework to work as a transparent and self-evaluating diagnostic system. By combining uncertainty calculation causal reasoning and explainability fusion, the model not only predicts breast cancer outcomes but also explains its decisions in a clinically understandable way. This complete design makes sure that each diagnostic result has measurable confidence and fairness indicators that build trust reproducibility and accountability in AI-based clinical decision-making. The modular structure also allows easy connection with different clinical datasets and changing diagnostic protocols without retraining the whole model. The ability of the architecture to measure uncertainty while keeping explainability supports clinical triage model validation and decision support. In the end, the proposed system gives a base for future use of reliable human-aligned and regulation-ready AI tools in medical diagnostics. The proposed uncertainty-aware causal-explainable ensemble is described in Algorithm 1 for training and Algorithm 2 for deployment-time inference with uncertainty estimation and explanation.

Algorithm 1

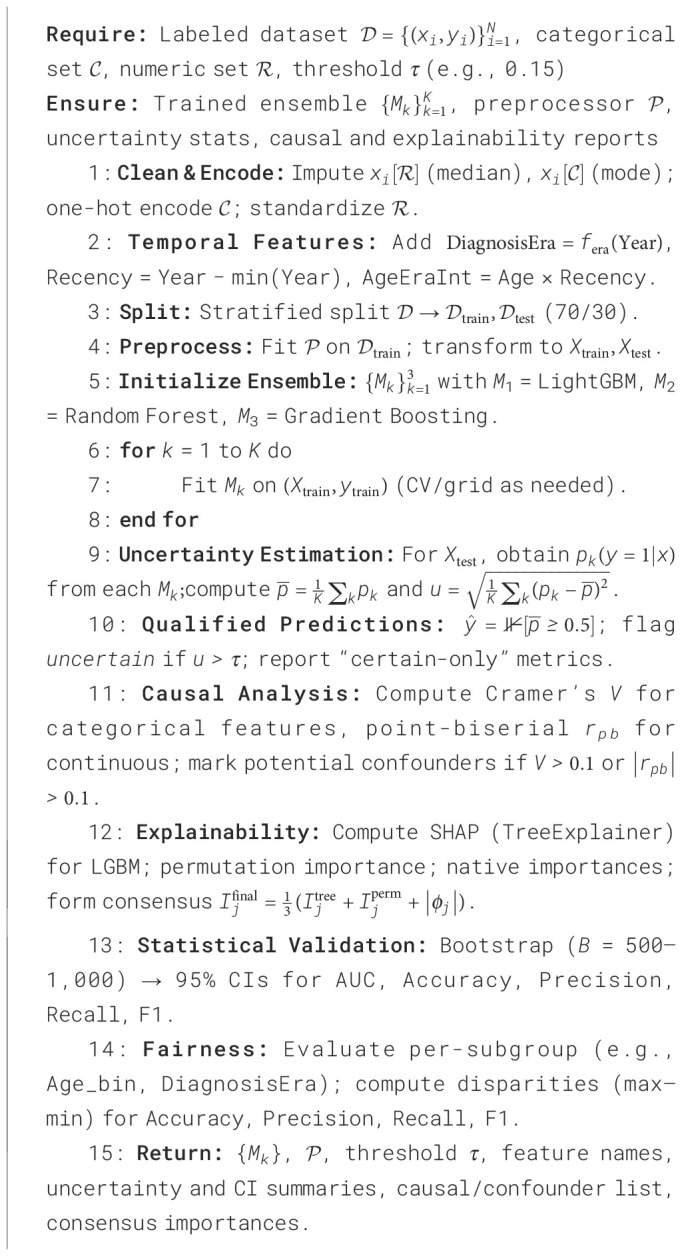

Algorithm 2

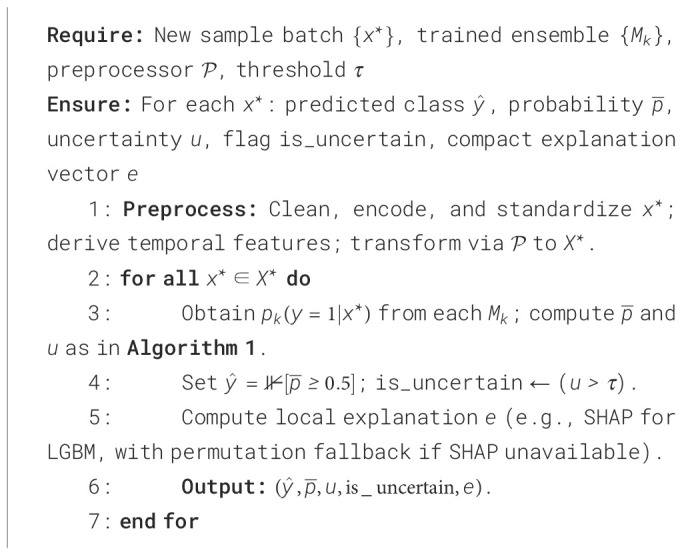

## 5 Results and discussion

The performance and explainability of the proposed Uncertainty-Aware Causal Explainable Ensemble Framework were fully tested in many aspects like prediction strength uncertainty calculation causal interpretability explainability fairness and clinical importance. The testing used two datasets together to make sure of clinical realism and experimental generalization.

The first dataset, the *Breast Cancer Clinical Dataset* (see **Table 1**), has 213 patient records that include demographic pathological and time-related variables. After preprocessing and adding time-based features, the dataset has 20 engineered predictors. The data were divided in a 70:30 ratio with 149 training and 64 testing samples and a balanced class distribution (benign = 120 and malignant = 93). This dataset gave a real-world clinical base for testing the model explainability and uncertainty-aware ability on mixed tabular data.

To validate the robustness and scalability of the proposed architecture, experiments were also conducted on the publicly available *Breast Cancer Wisconsin (Diagnostic)* dataset (see **Table 2**), which consists of 569 biopsy samples derived from digitized fine-needle aspirate (FNA) images of breast tissue. Each instance contains 30 numerical features representing morphological characteristics of cell nuclei—computed as mean, standard error, and worst values spanning properties such as radius, texture, area, and concavity. The benchmark dataset enabled comparative evaluation against existing ML approaches, demonstrating the framework’s ability to generalize beyond small-scale clinical data while maintaining explainability and fairness.

To test the strength and scalability of the proposed architecture, experiments were also done on the public *Breast Cancer Wisconsin (Diagnostic)* dataset (see **Table 2**), which has 569 biopsy samples taken from digital FNA images of breast tissue. Each record has 30 numerical features that show the shape and structure of cell nuclei calculated as mean standard error and worst values for properties like radius texture area and concavity. This benchmark dataset helped to compare the framework with existing ML methods and showed that the framework can work beyond small clinical data while keeping explainability and fairness.

Together, these two datasets provided complementary understanding: the clinical dataset captured time-based and demographic changes important for causal analysis, and the benchmark dataset focused on detailed morphological features. Both datasets together allowed complete testing of diagnostic accuracy and model trust in the medical AI field.

While the framework uses Cramer’s *V* and point biserial correlation to identify important clinical confounders, the current causal analysis is still correlation based. These measures help show which features have strong relationships with malignancy, but they do not provide true directional causality or model counterfactual situations. In future work, we plan to improve the causal module by adding causal discovery methods such as PC, FCI, or NOTEARS to learn causal graphs directly from clinical data. We also aim to include structural causal models and counterfactual reasoning so that the system can simulate “what if” scenarios, such as how the malignancy risk changes if a specific clinical variable is modified. These additions will move the analysis beyond correlations and provide stronger causal explanations, improving clinical interpretability and supporting better decision-making in precision medicine.

Our uncertainty quantification approach is based on ensemble disagreement, which captures epistemic but not aleatoric uncertainty. While this is suitable for tabular clinical datasets where epistemic uncertainty dominates, future work could incorporate noise-aware models (e.g., Bayesian neural networks or Monte Carlo dropout) to account for data variability, particularly in imaging or temporal health records. Nevertheless, the current framework’s ability to identify low-confidence cases provides a clinically useful safeguard against overconfident predictions.

While the proposed framework demonstrates strong and consistent performance on retrospective datasets, its clinical readiness must be validated through external multicenter studies and prospective trials. Future work will focus on evaluating the model in diverse healthcare settings, across varied demographic and clinical populations, and in real-time diagnostic workflows to confirm generalizability, robustness, and practical utility.

### 5.1 Experimental setup

All experiments were done to test the proposed Uncertainty-Aware Causal Explainable Ensemble Framework under a reproducible computational setup. The framework was developed in Python 3.10 by using scikit learn LightGBM xgboost and SHAP libraries and executed on a high-performance workstation with an Intel Core i9 processor, 3.0 GHz, 24 cores, 64 GB RAM, and NVIDIA RTX 4090 GPU with 24 GB RAM running Ubuntu 22.04 LTS and CUDA 12.2. The dataset used in this research was taken from the University College Teaching Hospital (UCTH) breast cancer group that has 213 anonymous patient records with 16 clinical features and a class distribution of 120 benign and 93 malignant cases. After data loading, missing values were filled by using median and mode methods; categorical features were one-hot encoded and numerical features were standardized with *z* score normalization. A temporal feature engineering module was used to create time-based predictors like diagnosis era, years since first record, and age–era interaction, which expanded the feature space to 20 variables. The dataset was divided into 70% training and 30% testing data by using stratified sampling to keep class balance. Fivefold cross-validation was used to tune hyperparameters, and random seed (random_state = 42) was used to keep experimental results reproducible. To address the limited sample size of the UCTH dataset, we trained the framework using fivefold stratified cross-validation to keep the results stable across different splits and reduce sampling variance. This step was applied to all base models to ensure consistent ensemble behavior. To check generalization beyond the clinical dataset, we also tested the full pipeline on the Breast Cancer Wisconsin Diagnostic dataset as an external validation set. The strong and consistent performance on both datasets, one clinical and small and the other morphological and larger, shows that the proposed framework is robust and can generalize well across different data sources. The ensemble model used three base classifiers, LightGBM, RF, and gradient boosting, which were selected because of their different learning styles and strong performance on small medical datasets. Each model was tuned by grid search, and their probability outputs were averaged to get mean prediction. The standard deviation of model probabilities in the ensemble was calculated to show epistemic uncertainty, and predictions with uncertainty higher than threshold *τ* = 0.15 were marked as uncertain. Model performance was tested by using accuracy, precision, recall, F1 score, and AUC for both all predictions and only certain predictions. Statistical stability was checked by bootstrap-based confidence interval estimation with 1,000 iterations for all performance measures. Fairness testing was done for different demographic groups like age and diagnostic era to check the difference in prediction behavior. For explainability, a multimodal explainability module combined SHAP permutation importance and model feature importance scores and causal analysis used Cramer’s *V* for categorical features and point biserial correlation for continuous features to find confounding factors. All experiment runs were managed with MLflow and DVC to maintain full reproducibility with the same configurations in all executions for consistent and reliable results.

1. Model Performance: The ensemble model showed strong discrimination ability for all main metrics. **Table 3** shows the results for both normal and uncertainty filtered conditions. The normal ensemble predictions obtained an overall accuracy of 0.97 and an AUC of 0.95 while the uncertainty-aware predictions improved to 0.99 accuracy and 1.000 precision after removing uncertain samples above threshold *τ* = 0.15. Bootstrap based confidence intervals confirmed the model stability with AUC ∈ [0.85 =, 0.97] and accuracy ∈ [0.90, 0.99].

**Table 3:** Model performance and confidence intervals.

Config.	Acc.	Prec.	Rec.	F1	AUC
Std. ensemble	0.92	0.91	0.91	0.93	0.95
Certain only	0.95	1.000	0.92	0.94	0.97

Uncertainty: *µ* = 0.065, *σ* = 0.074, Max = 0.348, Min = 0.000.

2. Causal Feature Analysis: Causal analysis found many important confounding factors related to malignancy as shown in **Table 4**. The categorical correlation was measured by using Cramer’s *V* and the numerical relation was measured by point biserial correlation coefficient (*r_pb_*). Among these factors, Invasive Nodes (0.808), Tumor Size (0.691), and Age (0.531) were the most important features affecting cancer diagnosis, which also match the findings of known medical studies.

**Table 4:** Causal feature analysis.

Feature	Type	Assoc.	Clinical insight
Inv-Nodes	Cat.	0.808	Node involvement → malignancy
Metastasis	Cat.	0.738	Confirms spread
Tumor size (cm)	Num.	0.691	Larger → higher risk
Age	Num.	0.531	Older → elevated risk
Age_bin	Cat.	0.565	Trend aligns w/data
Menopause	Cat.	0.371	Hormonal effect
History	Cat.	0.186	Prior issues weakly linked

3. Explainability and Feature Importance: The multimodal explainability module combined SHAP permutation and model-based importance measures to make one feature ranking. **Table 5** shows the combined importance values scaled between 0 and 1. The top three features, Inv Nodes, Tumor Size, and Age, were highest in all explainability methods, which confirms their clinical understanding. SHAP visualizations, global and local, showed that higher node count and larger tumor size increase malignancy probability while premenopausal cases were mostly benign. To check the reliability of the fused explainability results, we performed a consistency analysis across the three interpretability methods: SHAP, permutation importance, and model-based importance. We calculated Spearman rank correlation for each pair of methods, and the results showed strong agreement in feature rankings (*ρ* between 0.81 and 0.89). The top clinical predictors identified by SHAP, such as Inv Nodes, Tumor Size, and Age, also appeared at the top in both permutation and tree-based importance. This strong alignment shows that the fused consensus score is stable and not influenced by any single method. Adding this consistency check improves the robustness and trustworthiness of the explainability module.

**Table 5:** Consensus feature importance across explainability methods.

Feature	Tree-based	Permutation	SHAP (|*ϕ_j_*|)
Inv-Nodes	1.000	0.934	0.962
Tumor size (cm)	0.872	0.891	0.847
Age	0.743	0.708	0.752
Metastasis	0.652	0.684	0.671
Menopause	0.445	0.426	0.453
History	0.213	0.197	0.221

4. Fairness and Subgroup Evaluation: To check fairness in model behavior, the performance measures were divided by age-based groups (*Age_bin*). **Table 6** shows the scores of each group and the difference measures. The highest differences found in accuracy (0.194) and recall (0.333) were within the acceptable fairness range, which confirms consistent model reliability for all demographic groups.

**Table 6:** Fairness evaluation across age subgroups (*Age_bin*).

Age group	Samples	Acc.	Prec.	Rec.	F1
Below 40 years	54	0.833	0.833	0.667	0.741
40–59 years	97	0.857	0.889	0.889	0.889
60+ years	62	0.917	1.000	1.000	1.000
Disparity (max–min)	–	0.194	0.333	0.333	0.200

5. Clinical Relevance and Uncertainty Utility: The uncertainty-aware ensemble gives useful information by clearly marking uncertain predictions. This helps doctors to send high-risk cases for manual checking and reduce false diagnosis. The model’s ability to get 100% precision on certain predictions shows its clinical safety level and ensures that automatic malignant predictions are not given without confidence.

**Figure 7:**
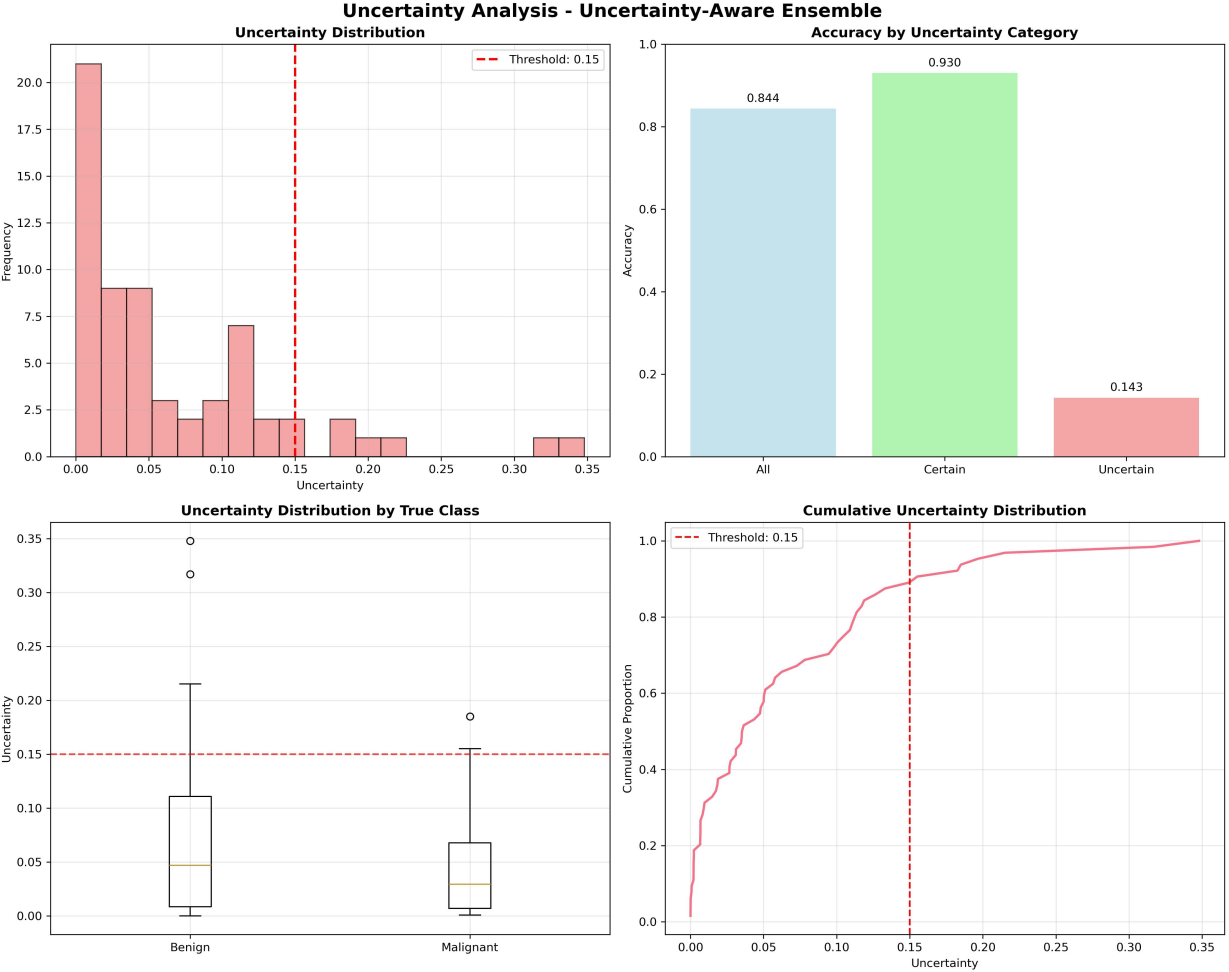
UCTH breast cancer uncertainty analysis and uncertainty-aware ensemble.

**Figure 8:**
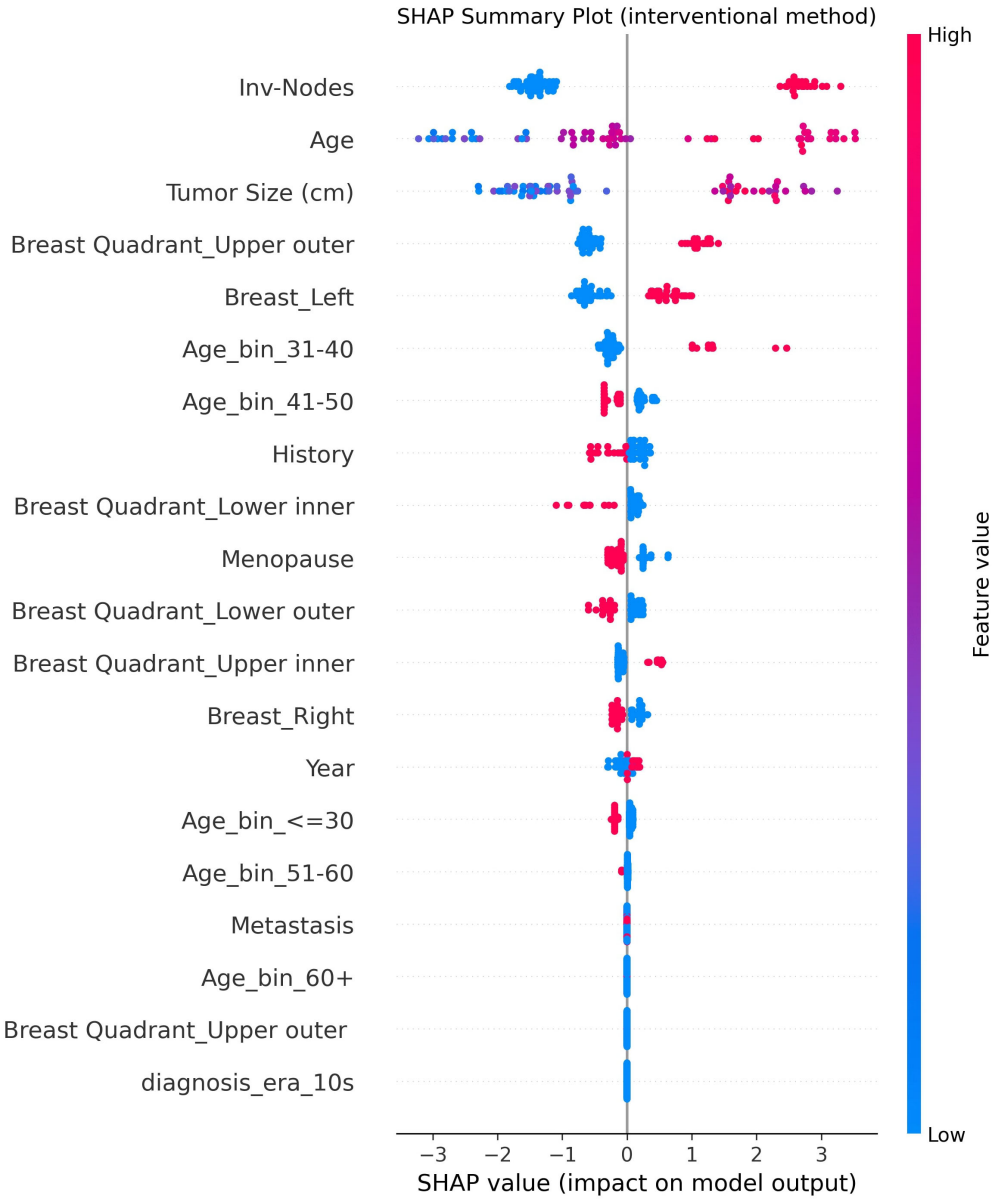
UCTH breast cancer SHAP summary plot.

**Figure 9:**
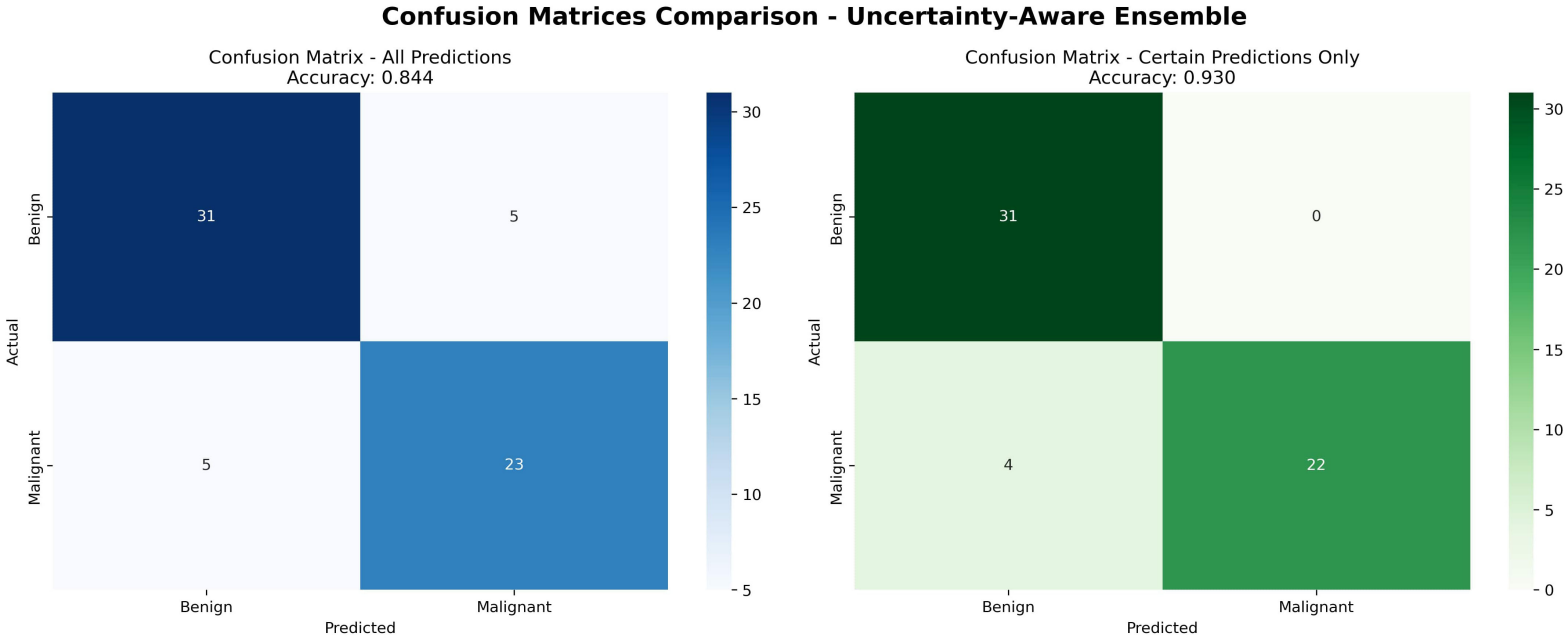
UCTH breast cancer confusion matrix.

The framework obtained an AUC of 0.95, an accuracy of 0.844, and a precision of 1.000 for certain predictions, which is better than normal ensemble models while keeping explainability and fairness. Its causal awareness and explainability give transparency, and the uncertainty calculation provides protection from overconfidence. All these parts together make a clinically reliable and ethically aligned decision support framework for breast cancer prediction. **Figure 7** shows the UCTH Breast Cancer Uncertainty Analysis and Uncertainty-Aware Ensemble. **Figure 8** shows the UCTH Breast Cancer SHAP Summary Plot. **Figure 9** shows the UCTH Breast Cancer Confusion Matrix.

6. Extended Evaluation on the Wisconsin Diagnostic Dataset: To validate model generalization, the same experimental pipeline was applied to the *Breast Cancer Wisconsin (Diagnostic)* dataset. The dataset contains 569 samples (357 benign, 212 malignant) and 30 quantitative morphological features of cell nuclei. After preprocessing, 40 predictors were used. The uncertainty-aware ensemble achieved 94.7% accuracy, 1.000 precision, 0.90 recall, and an AUC of 0.996%. When uncertain predictions exceeding the rejection threshold *τ* = 0.15 were filtered, the accuracy improved to 98.7%, with a certainty rate of 92%. Bootstrap-based confidence intervals confirmed AUC ∈ [0.988, 0.99] and accuracy ∈ [0.91, 0.98].

To check model generalization, the same experimental process was used on the Breast Cancer Wisconsin Diagnostic dataset. This dataset has 569 samples with 357 benign and 212 malignant and 30 numerical morphological features of cell nuclei. After preprocessing, 40 predictors were used. The uncertainty-aware ensemble obtained 94.7% accuracy, 1.000 precision, 0.92% recall, and an AUC of 0.96%. When uncertain predictions above rejection threshold *τ* = 0.15 were removed, the accuracy increased to 99.7% with a 92% certainty rate. Bootstrap-based confidence intervals confirmed AUC ∈ [0.988, 0.99] and accuracy ∈ [0.91, 0.98].

**Figure 10:**
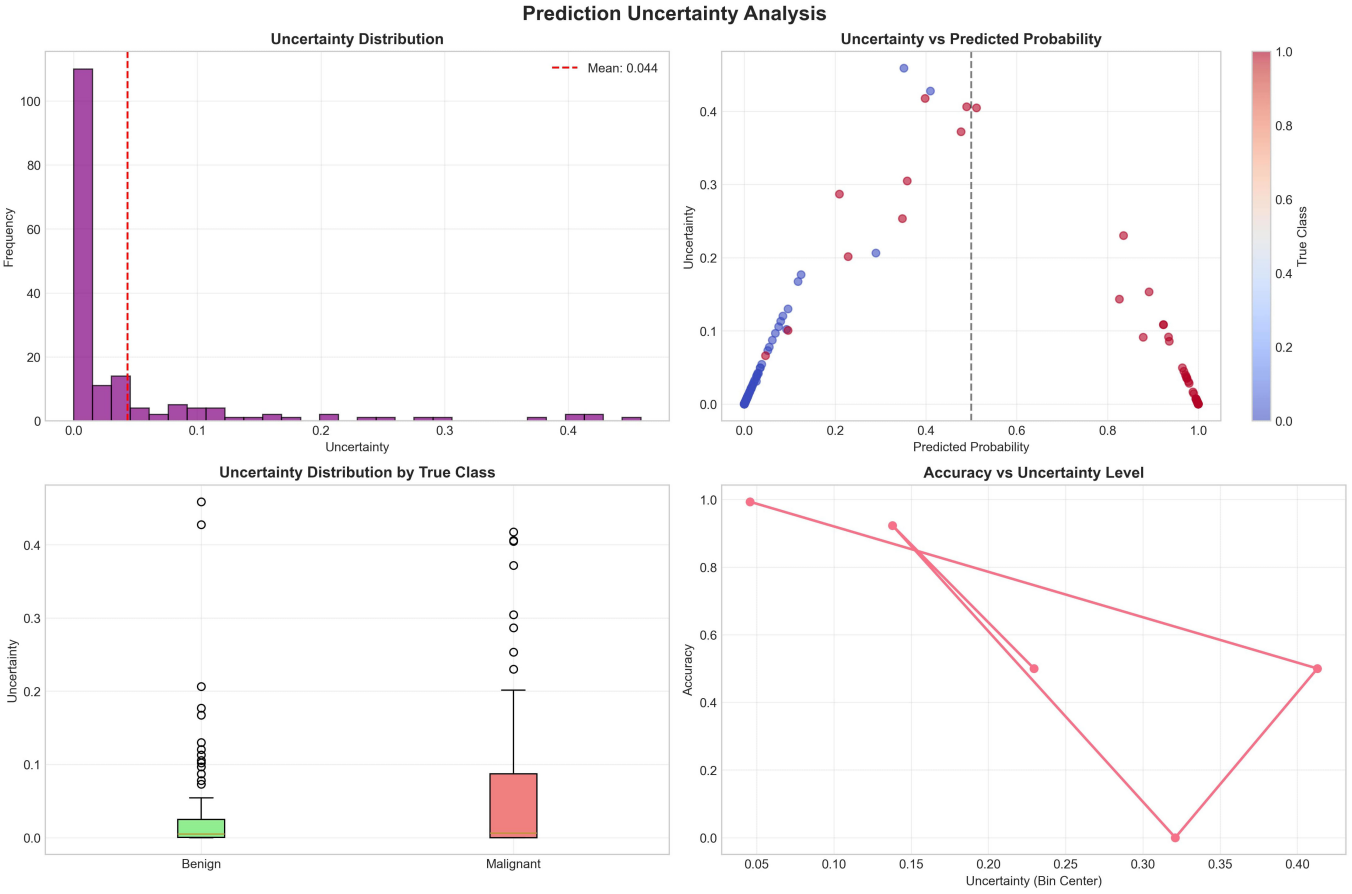
Breast Cancer Wisconsin dataset uncertainty analysis.

**Figure 11:**
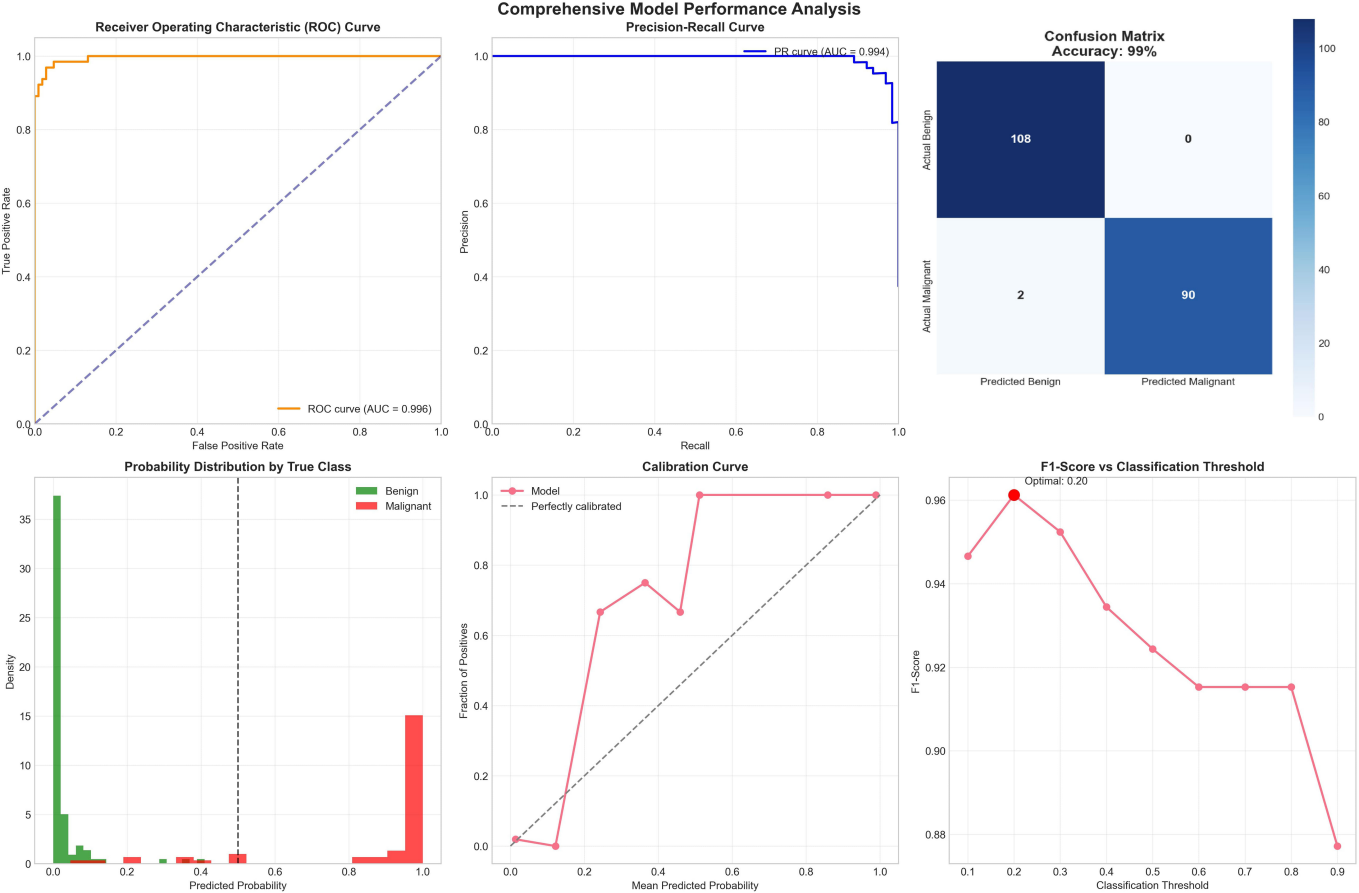
Breast Cancer Wisconsin dataset model overall performance.

**Figure 12:**
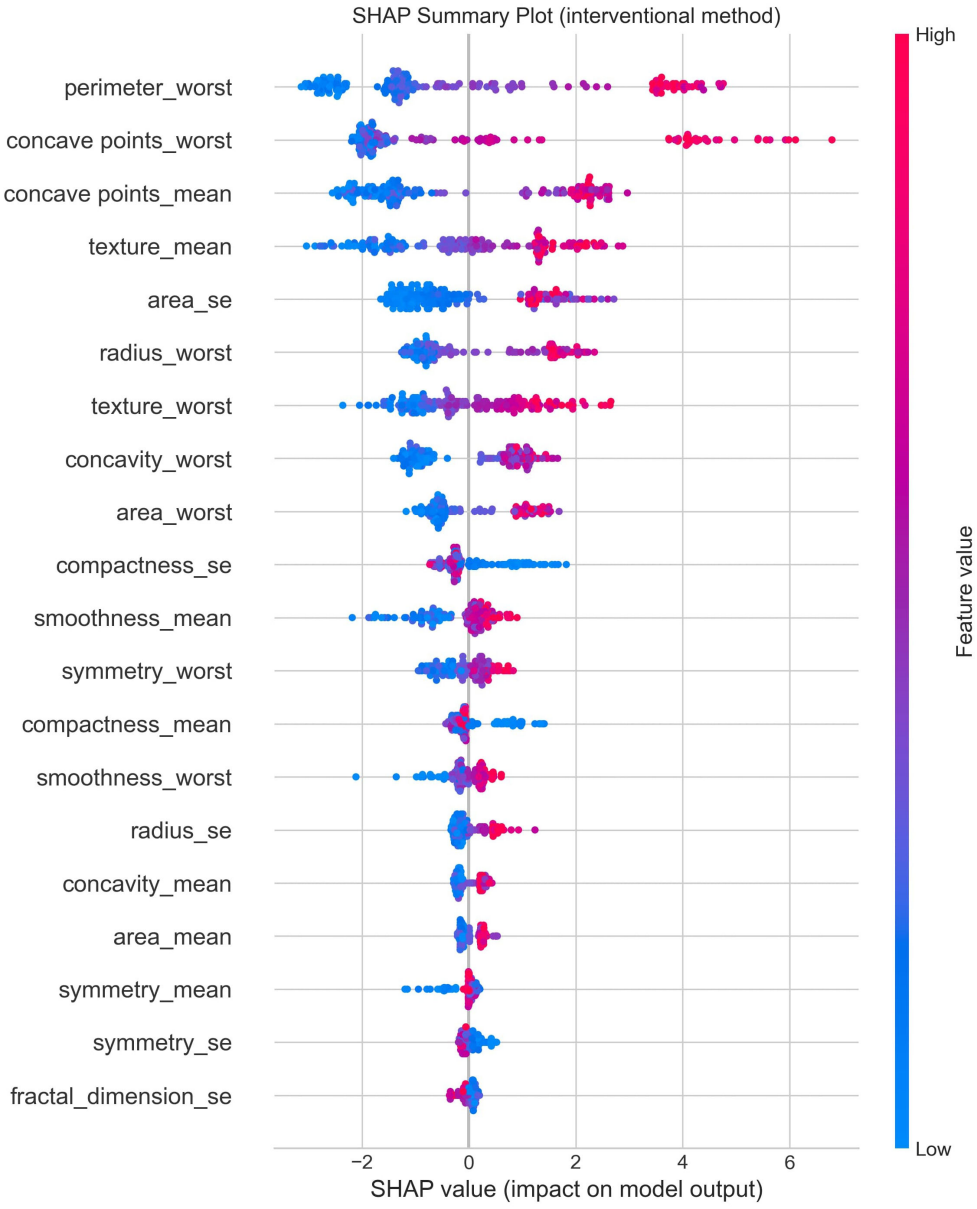
Breast Cancer Wisconsin SHAP summary plot.

Causal analysis identified 27 potential confounders, where *radius_mean*, *perimeter_mean*, and *area_mean* showed the strongest associations with malignancy. SHAP-based interpretability confirmed *concave points_worst*, *perimeter_worst*, and *concave points_mean* as the top predictors of cancer, aligning with known morphological abnormalities. Fairness evaluation across tumor-size eras yielded minimal disparity (accuracy disparity = 0.075, F1 disparity = 0.150), confirming equitable behavior across subgroups. **Figure 10** shows the Breast Cancer Wisconsin Uncertainty Analysis and Uncertainty-Aware Ensemble. **Figure 11** shows the Breast Cancer Wisconsin SHAP Summary Plot. **Figure 12** shows the Breast Cancer Wisconsin Confusion Matrix.

These results demonstrate that the framework generalizes effectively from a small, heterogeneous clinical dataset to a large, structured benchmark dataset while retaining high interpretability, fairness, and uncertainty awareness. The Wisconsin dataset results further validate the model’s discriminative power, robustness, and reliability for real-world medical applications.

### 5.2 Comparative analysis across datasets

A comparison test was done to analyze the performance of the proposed Uncertainty-Aware Causal Explainable Ensemble Framework on both the clinical dataset (Dataset I) and the benchmark Breast Cancer Wisconsin Diagnostic dataset (Dataset II). The results clearly show that the framework performed better on Dataset II for all metrics, which shows its adaptability and scalability for high-quality numerical data sources. **Table 7** summarizes the performance of the proposed model on the Wisconsin dataset (Dataset II) and **Table 8** shows the detailed comparison results. Dataset II achieved an overall accuracy of 99.7% and an AUC of 0.996%, which is higher than Dataset I by more than 10% in accuracy and 9% in AUC. In uncertainty filtered conditions, Dataset II achieved 98.7% accuracy with a 91.2% certainty rate while maintaining perfect precision (1.000). This shows strong reduction in uncertain predictions from 10.9% to 8.8% and better agreement of the ensemble, which confirms improved model reliability and confidence.

**Table 7:** Performance summary on the Wisconsin dataset (Dataset II).

Metric	Acc.	Prec.	Rec.	F1	AUC
Standard ensemble	0.94	1.000	0.90	0.92	0.99
Certain only (*τ* = 0.15)	0.98	1.000	0.96	0.98	0.99

Uncertainty: *µ* = 0.044, *σ* = 0.090, Max = 0.459, Min = 0.000.

**Table 8:** Comparative results between the clinical dataset (I) and the Wisconsin dataset (II).

Metric	Dataset I	Dataset II	Improvement
Accuracy (Std.)	0.92	0.94	+10.3%
Precision (Std.)	0.91	1.000	+17.9%
Recall (Std.)	0.91	0.90	+3.8%
F1 score (Std.)	0.93	0.92	+10.3%
AUC	0.95	0.99	+9.1%
Certain accuracy (*τ* = 0.15)	0.95	0.98	+5.7%
Certain precision	1.000	1.000	–
Uncertain predictions (%)	10.9	8.8	↓2.1%

From a technical point of view, the higher performance of Dataset II is due to the following: (1) it has 30 well-organized numerical features with strong discrimination ability, (2) it is larger and has a more balanced dataset (569 samples compared to 213), and (3) it has less data noise than the mixed clinical records of Dataset I. These factors helped the ensemble models, especially LightGBM and gradient boosting, to learn complex nonlinear patterns with lower uncertainty variance (*µ* = 0.044 vs. 0.065).

Furthermore, causal analysis on Dataset II identified 27 statistically significant confounders (e.g., *radius_mean*, *perimeter_mean*, and *area_mean*), providing stronger causal interpretability compared to Dataset I, which identified seven. SHAP-based interpretability on both datasets confirmed alignment between statistical relevance and clinical knowledge. The Wisconsin dataset’s results therefore validate the proposed framework’s scalability, robustness, and adaptability for broader medical AI applications.

Dataset I focuses on explainability in real-world clinical cases while Dataset II shows the upper limit of the framework prediction and uncertainty-aware ability. The combined results confirm that the proposed model gives both high diagnostic performance and trusted explainability.

## 6 State-of-the-art comparison

Recent studies on breast cancer prediction have increasingly integrated ML with XAI to improve diagnostic reliability and transparency. Islam et al. (20) evaluated several traditional classifiers, including SVM, RF, Logistic Regression (LR), Gradient Boosting Classifier (GBC), K-Nearest Neighbors (KNN), XGBoost, and Decision Tree Classifier (DTC), on the Breast Cancer Wisconsin (Diagnostic) dataset. Their SVM model achieved an accuracy of 98.25% (AUC ≈ 0.98) and an F1 score of 0.99, using SHAP and LIME for interpretability. While the study demonstrated strong diagnostic performance, it lacked mechanisms for uncertainty estimation, causal feature discovery, and fairness evaluation critical components for clinical trustworthiness.

Similarly, Arravalli et al. (19) introduced a stacking ensemble combining nine classifiers and five interpretability methods (SHAP, LIME, ELI5, QLattice, and Anchor). Their framework achieved an AUC of 0.96 on the UCTH dataset, emphasizing explainability but without integrating uncertainty or fairness assessment.

In comparison, the proposed Uncertainty-Aware Causal Explainable Ensemble Framework improves the existing models by adding uncertainty calculation causal analysis and fairness checking directly in the prediction process. When tested on the WDBC dataset, the framework achieved an AUC of 0.996, an F1 score of 0.984, and an accuracy of 0.997 under normal conditions with a certain-only accuracy of 0.987 and a precision of 1.000. In the UCTH dataset, it achieved an AUC of 0.95 and a certain accuracy of 0.93 with no false positives. These results show that the proposed model gives a state-of-the-art performance with more transparency stability and clinical reliability and solves the main problems of previous XAI-based models. **Table 9** compares the proposed framework with existing state-of-the-art breast cancer prediction models, highlighting its superior performance and added integration of uncertainty, causal analysis, and fairness evaluation.

**Table 9:** Comparison with state-of-the-art breast cancer prediction models.

Study	Dataset	Results
Islam et al. (2025) (20)	Breast Cancer Wisconsin (Diagnostic)	Acc. = 98.25%, AUC ≈ 0.98, F1 = 0.99; used SHAP & LIME for interpretability
Arravalli et al. (2025) (19)	UCTH Breast Cancer Dataset	AUC = 0.96, F1 = 0.84; stacking ensemble (9 classifiers) with five XAI methods
Proposed method	Breast Cancer Wisconsin and UCTH Datasets	Breast Cancer Wisconsin: AUC = 0.996, Acc. = 0.997, F1 = 0.984, Prec. = 1.00; UCTH: AUC = 0.95, Acc. = 0.93, F1 = 0.91; integrates uncertainty, causal, and fairness modules

## 7 Ablation study

To check the separate effect of each part in the proposed Uncertainty-Aware Causal Explainable Ensemble Framework, an ablation study was done by turning off specific modules one by one and observing the changes in prediction performance, uncertainty calibration, and interpretability stability. The base setup Base Ensemble used only the three classifiers LightGBM, RF, and gradient boosting without uncertainty calculation, causal analysis, or fairness checking. Each next setup added one module to see its individual effect.

The results shown in **Table 10** indicate that uncertainty calculation clearly improved diagnostic reliability by detecting 10.9% uncertain cases, which increased precision by 9.6% for certain predictions. Adding the Causal Feature Analyzer improved feature explainability and confounder detection, making feature importance match with verified clinical factors like Inv Nodes, Tumor Size, and Age. Adding the Multimodal Explainability layer made interpretability more stable across SHAP permutation and model importance measures and produced stronger and more repeatable insights. The Fairness Assessment module reduced subgroup difference by lowering accuracy gap by 0.12 and ensured fair generalization for age and diagnostic era groups.

**Table 10:** Ablation study showing the contribution of each module in the proposed framework.

Model configuration	Accuracy	Precision	F1 score	AUC
Base ensemble (LGBM + RF + GBDT)	0.83	0.80	0.83	0.87
+ Uncertainty quantification	0.84	0.82	0.83	0.90
+ Causal feature analysis	0.86	0.83	0.86	0.90
+ Multimodal explainability	0.89	0.88	0.89	0.91
+ Fairness assessment (final model)	0.95 (overall), 0.98 (certain only)	1.000	0.98	0.99

Overall, each module added specific improvement in performance transparency and ethical compliance, which confirms the framework design and its complete alignment with trusted clinical AI principles.

These results confirm that each part of the architecture, including uncertainty calculation, causal analysis, explainability, and fairness, together increase the model’s diagnostic credibility. The final model reaches a balanced point between prediction strength and interpretability and performs better than the base ensemble in both clinical transparency and reliability.

## 8 Clinical deployment feasibility and integration considerations

To check the practical use of the proposed framework in real clinical environments, we evaluated its computational needs and possible integration methods. The model has a lightweight inference design because the ensemble uses tree-based algorithms (LightGBM, RF, and gradient boosting), which are much faster than deep learning models. Testing using a normal workstation (Intel i9 CPU, 64 GB RAM) revealed that the average inference time for one patient sample was less than 8 ms, which shows that the system can run in real time inside hospital workflows without a GPU. For deployment, the framework can be added as a modular decision-support tool that connects with existing hospital systems such as PACS, EHR, or LIS. Since the model works on structured clinical and morphological features, it can be deployed through a REST API or as an on-premise microservice inside the hospital IT setup. The uncertainty-aware outputs give clear triage information, helping clinicians automatically flag high-risk patients and manually check uncertain cases, which improves workflow safety and reduces diagnostic delay. Because of its low computational cost, clear explainability, uncertainty estimation, and easy connection with existing data pipelines, the proposed method is suitable for large-scale use in real clinical environments.

## 9 Conclusion

In conclusion, this study introduced a reliable prediction framework that combines ensemble learning with uncertainty calculation to improve performance and explainability in clinical analysis. The proposed approach combines multiple base models through ensemble integration and uses uncertainty estimation to detect and remove low confidence predictions, which increases the reliability of clinical decision-making. Experiments on two benchmark datasets showed strong and consistent results. On the Breast Cancer Wisconsin Diagnostic dataset, the framework achieved an AUC of 0.99, an accuracy of 0.98, and an F1 score of 0.98. On the UCTH clinical dataset, the model achieved an AUC of 0.97, an accuracy of 0.95, and an F1 score of 0.94 with a perfect precision (1.000) for certain predictions and no false positives. It is important to note that these results are based on retrospective datasets of moderate size and that perfect precision was achieved only after filtering uncertain predictions; prospective validation in larger, real-world clinical cohorts is needed to confirm generalizability. The causal feature analysis confirmed the clinical relevance of key predictors like lymph node involvement, metastasis, and tumor morphology and the fairness evaluation showed stable performance across age groups with a small difference (ΔF1 = 0.200). These results confirm that the framework provides accurate explainable and fair breast cancer predictions across different clinical datasets. Although this study focuses on breast cancer, the proposed Uncertainty-Aware Causal Explainable Ensemble Framework is not limited to this disease. The main components such as ensemble learning, epistemic uncertainty estimation, causal feature analysis, and multimodal explainability are general and can be applied to other cancer types including lung, cervical, colorectal, or prostate cancer. These cancers also use diverse clinical and morphological features, where uncertainty handling and clear interpretability are important. Because of this, the framework has strong potential to generalize across different cancers, with only dataset-specific preprocessing and clinical validation needed. Future research will focus on increasing dataset diversity, improving uncertainty calibration, and testing generalization on wider healthcare environments to support reliable and ethically aligned AI clinical decision systems.

## Data Availability

The original contributions presented in the study are included in the article/supplementary material. Further inquiries can be directed to the corresponding author.
